# Hospitalization of Adolescents Aged 12–17 Years with Laboratory-Confirmed COVID-19 — COVID-NET, 14 States, March 1, 2020–April 24, 2021

**DOI:** 10.15585/mmwr.mm7023e1

**Published:** 2021-06-11

**Authors:** Fiona P. Havers, Michael Whitaker, Julie L. Self, Shua J. Chai, Pam Daily Kirley, Nisha B. Alden, Breanna Kawasaki, James Meek, Kimberly Yousey-Hindes, Evan J. Anderson, Kyle P. Openo, Andrew Weigel, Kenzie Teno, Maya L. Monroe, Patricia A. Ryan, Libby Reeg, Alexander Kohrman, Ruth Lynfield, Kathryn Como-Sabetti, Mayvilynne Poblete, Chelsea McMullen, Alison Muse, Nancy Spina, Nancy M. Bennett, Maria Gaitán, Laurie M. Billing, Jess Shiltz, Melissa Sutton, Nasreen Abdullah, William Schaffner, H. Keipp Talbot, Melanie Crossland, Andrea George, Kadam Patel, Huong Pham, Jennifer Milucky, Onika Anglin, Dawud Ujamaa, Aron J. Hall, Shikha Garg, Christopher A. Taylor, COVID-NET Surveillance Team, COVID-NET Surveillance Team, Gretchen Rothrock, Arthur Reingold, Millen Tsegaye, Sarah McLafferty, Amber Maslar, Paula Clogher, Adam Misiorski, Christina Parisi, Maria Correa, Tessa Carter, Carol Lyons, Daewi Kim, Gaggan Brar, Emily Fawcett, Allison Roebling, Katelyn Ward, Jana Manning, Asmith Joseph, Chandler Surell, Daniel Pizarro, Jeremiah Williams, Rayna Ceaser, Stephanie Lehman, Taylor Eisenstein, Gracie Chambers, Grayson Kallas, Lauren Russell, Suzanne Segler, David Blythe, Alicia Brooks, Erica Bye, Richard Danila, Cory Cline, Susan Ropp, Chad Smelser, Daniel Sosin, Salina Torres, Kathy Angeles, Melissa Christian, Nancy Eisenberg, Kristina Flores, Caroline Habrun, Emily Hancock, Sarah Khanlian, Meaghan Novi, Erin Phipps, Dominic Rudin, Yadira Salazar-Sanchez, Judith Segall, Sarah Shrum Davis, Grant Barney, Christina Felsen, Sophrena Bushey, Kevin Popham, Virginia Cafferky, Christine Long, RaeAnne Kurtz, Nicole West, Ama Owusu-Dommey, Breanna McArdle, Emily Youngers, Kylie Seeley, Tiffanie Markus, Amanda Carter, Andrea Price, Andrew Haraghey, Ashley Swain, Caitlin Shaw, Ian Buchta, Jake Ortega, Laine McCullough, Ryan Chatelain, Tyler Riedesel

**Affiliations:** ^1^CDC COVID-19 Response Team; ^2^California Emerging Infections Program, Oakland, California; ^3^Career Epidemiology Field Officer Program, CDC; ^4^Colorado Department of Public Health and Environment, ^5^Connecticut Emerging Infections Program, Yale School of Public Health, New Haven, Connecticut; ^6^Departments of Medicine and Pediatrics, Emory University School of Medicine, Atlanta, Georgia; ^7^Georgia Emerging Infections Program, Georgia Department of Public Health; ^8^Atlanta Veterans Affairs Medical Center, Atlanta, Georgia; ^9^Division of Infectious Diseases, School of Medicine, Emory University, Atlanta Georgia; ^10^Iowa Department of Public Health; ^11^Maryland Department of Health; ^12^Michigan Department of Health and Human Services; ^13^Minnesota Department of Health; ^14^New Mexico Emerging Infections Program, University of New Mexico, Albuquerque, New Mexico; ^15^New Mexico Emerging Infections Program, New Mexico Department of Health; ^16^New York State Department of Health; ^17^University of Rochester School of Medicine and Dentistry, Rochester, New York; ^18^Ohio Department of Health; ^19^Public Health Division, Oregon Health Authority; ^20^Vanderbilt University Medical Center, Nashville, Tennessee; ^21^Salt Lake County Health Department, Salt Lake City, Utah; ^22^General Dynamics Information Technology, Atlanta, Georgia; ^23^Influenza Division, National Center for Immunization and Respiratory Diseases, CDC.; California Emerging Infections Program, Oakland, California; University of California; Colorado Department of Public Health & Environment; Colorado Department of Public Health & Environment; , Connecticut Emerging Infections Program; Connecticut Emerging Infections Program; Connecticut Emerging Infections Program; Connecticut Emerging Infections Program; Connecticut Emerging Infections Program; Connecticut Emerging Infections Program; Connecticut Emerging Infections Program; Connecticut Emerging Infections Program; Connecticut Emerging Infections Program; Georgia Emerging Infections Program, Georgia Department of Public Health, Veterans Affairs Medical Center, Foundation for Veterans Education and Research; Georgia Emerging Infections Program, Georgia Department of Public Health, Veterans Affairs Medical Center, Foundation for Veterans Education and Research; Georgia Emerging Infections Program, Georgia Department of Public Health, Veterans Affairs Medical Center, Foundation for Veterans Education and Research; Georgia Emerging Infections Program, Georgia Department of Public Health, Veterans Affairs Medical Center, Foundation for Veterans Education and Research; Georgia Emerging Infections Program, Georgia Department of Public Health, Veterans Affairs Medical Center, Foundation for Veterans Education and Research; Georgia Emerging Infections Program, Georgia Department of Public Health, Veterans Affairs Medical Center, Foundation for Veterans Education and Research; Georgia Emerging Infections Program, Georgia Department of Public Health, Veterans Affairs Medical Center, Foundation for Veterans Education and Research; Georgia Emerging Infections Program, Georgia Department of Public Health, Veterans Affairs Medical Center, Foundation for Veterans Education and Research; Georgia Emerging Infections Program, Georgia Department of Public Health, Veterans Affairs Medical Center, Foundation for Veterans Education and Research; Georgia Emerging Infections Program, Georgia Department of Public Health, Veterans Affairs Medical Center, Foundation for Veterans Education and Research; Georgia Emerging Infections Program, Georgia Department of Public Health, Veterans Affairs Medical Center, Foundation for Veterans Education and Research; Georgia Emerging Infections Program, Georgia Department of Public Health, Veterans Affairs Medical Center, Foundation for Veterans Education and Research; Division of Infectious Diseases, School of Medicine, Emory University, Emerging Infections Program, Georgia Department of Health; Division of Infectious Diseases, School of Medicine, Emory University, Emerging Infections Program, Georgia Department of Health; Division of Infectious Diseases, School of Medicine, Emory University, Emerging Infections Program, Georgia Department of Health; Maryland Department of Health; Maryland Department of Health; Minnesota Department of Health; Minnesota Department of Health; New Mexico Department of Health; New Mexico Department of Health; New Mexico Department of Health; New Mexico Department of Health; New Mexico Department of Health; New Mexico Emerging Infections Program; New Mexico Emerging Infections Program; New Mexico Emerging Infections Program; New Mexico Emerging Infections Program; New Mexico Emerging Infections Program; New Mexico Emerging Infections Program; New Mexico Emerging Infections Program; New Mexico Emerging Infections Program; New Mexico Emerging Infections Program; New Mexico Emerging Infections Program; New Mexico Emerging Infections Program; New Mexico Emerging Infections Program; New Mexico Emerging Infections Program; New York State Department of Health; University of Rochester School of Medicine and Dentistry, Rochester, New York; University of Rochester School of Medicine and Dentistry, Rochester, New York; University of Rochester School of Medicine and Dentistry, Rochester, New York; University of Rochester School of Medicine and Dentistry, Rochester, New York; University of Rochester School of Medicine and Dentistry, Rochester, New York; University of Rochester School of Medicine and Dentistry, Rochester, New York; Public Health Division, Oregon Health Authority; Public Health Division, Oregon Health Authority; Public Health Division, Oregon Health Authority; Public Health Division, Oregon Health Authority; Oregon Health & Science University School of Medicine, Portland, Oregon; Vanderbilt University Medical Center; Salt Lake County Health Department, Utah; Salt Lake County Health Department, Utah; Salt Lake County Health Department, Utah; Salt Lake County Health Department, Utah; Salt Lake County Health Department, Utah; Salt Lake County Health Department, Utah; Salt Lake County Health Department, Utah; Salt Lake County Health Department, Utah; Salt Lake County Health Department, Utah; Salt Lake County Health Department, Utah

Most COVID-19–associated hospitalizations occur in older adults, but severe disease that requires hospitalization occurs in all age groups, including adolescents aged 12–17 years (*1*). On May 10, 2021, the Food and Drug Administration expanded the Emergency Use Authorization for Pfizer-BioNTech COVID-19 vaccine to include persons aged 12–15 years, and CDC’s Advisory Committee on Immunization Practices recommended it for this age group on May 12, 2021.* Before that time, COVID-19 vaccines had been available only to persons aged ≥16 years. Understanding and describing the epidemiology of COVID-19–associated hospitalizations in adolescents and comparing it with adolescent hospitalizations associated with other vaccine-preventable respiratory viruses, such as influenza, offers evidence of the benefits of expanding the recommended age range for vaccination and provides a baseline and context from which to assess vaccination impact. Using the Coronavirus Disease 2019-Associated Hospitalization Surveillance Network (COVID-NET), CDC examined COVID-19–associated hospitalizations among adolescents aged 12–17 years, including demographic and clinical characteristics of adolescents admitted during January 1–March 31, 2021, and hospitalization rates (hospitalizations per 100,000 persons) among adolescents during March 1, 2020–April 24, 2021. Among 204 adolescents who were likely hospitalized primarily for COVID-19 during January 1–March 31, 2021, 31.4% were admitted to an intensive care unit (ICU), and 4.9% required invasive mechanical ventilation; there were no associated deaths. During March 1, 2020–April 24, 2021, weekly adolescent hospitalization rates peaked at 2.1 per 100,000 in early January 2021, declined to 0.6 in mid-March, and then rose to 1.3 in April. Cumulative COVID-19–associated hospitalization rates during October 1, 2020–April 24, 2021, were 2.5–3.0 times higher than were influenza-associated hospitalization rates from three recent influenza seasons (2017–18, 2018–19, and 2019–20) obtained from the Influenza Hospitalization Surveillance Network (FluSurv-NET). Recent increased COVID-19–associated hospitalization rates in March and April 2021 and the potential for severe disease in adolescents reinforce the importance of continued COVID-19 prevention measures, including vaccination and correct and consistent wearing of masks by persons not yet fully vaccinated or when required by laws, rules, or regulations.^†^

COVID-NET is a population-based surveillance system of laboratory-confirmed COVID-19–associated hospitalizations in 99 counties across 14 states,^§^ covering approximately 10% of the U.S. population.^¶^ Included in surveillance are COVID-19–associated hospitalizations among residents in a predefined surveillance catchment area who had a positive real-time reverse transcription–polymerase chain reaction or rapid antigen detection test result for SARS-CoV-2 (the virus that causes COVID-19) during hospitalization or ≤14 days before admission (*2*). Clinical and demographic data, updated monthly, were analyzed for adolescents aged 12–17 years hospitalized during January 1–March 31, 2021. Clinical and demographic characteristics were analyzed separately for patients whose primary reason for admission was likely COVID-19 and those whose primary reason for admission might not have been primarily related to COVID-19, despite receiving a positive SARS-CoV-2 laboratory test result.** Hospitalization rate data, updated weekly, were analyzed during March 1, 2020–April 24, 2021, to describe cumulative COVID-19–associated hospitalization rates in adolescents aged 12–17 years and adults aged ≥18 years and weekly COVID-19–associated hospitalization rates in children aged 0–4 years and 5–11 years and adolescents aged 12–17 years. In addition, cumulative COVID-19–associated hospitalization rates among adolescents aged 12–17 years during October 1, 2020–April 24, 2021 (covering most of the typical October 1–April 30 season for influenza-associated hospitalization surveillance), were compared with influenza-associated hospitalization rates in the same age group across three influenza seasons (2017–18, 2018–19, and 2019–20) using data from FluSurv-NET^††^ (*3*). Rate calculations are unadjusted and include all persons meeting the case definition (*2*). SAS statistical software (version 9.4; SAS Institute) was used for analyses. This activity was reviewed by CDC and was conducted consistent with applicable federal law and CDC policy.^§§^

Among 376 adolescents hospitalized during January 1–March 31, 2021, who received a positive SARS-CoV-2 laboratory test result, 172 (45.7%) were analyzed separately because their primary reason for admission might not have been directly COVID-19–related (Table). Among the 204 patients who were likely admitted primarily for COVID-19–related illness, 52.5% were female, 31.4% were Hispanic or Latino (Hispanic), and 35.8% were non-Hispanic Black. Overall, 70.6% had one or more underlying medical conditions, the most common of which were obesity (35.8%), chronic lung disease, including asthma (30.9%), and neurologic disorders (14.2%); 31.4% of patients required ICU admission and 4.9% required invasive mechanical ventilation, but there were no associated deaths.

**TABLE:** Demographic and clinical characteristics and outcomes among adolescents aged 12–17 years with laboratory-confirmed COVID-19–associated hospitalizations, by primary reason for admission — COVID-NET, 14 states,* January 1, 2021–March 31, 2021

Characteristic	No. of hospitalizations (%)		
	Total	Primary reason for admission COVID-19–related	Primary reason for admission not clearly COVID-19–related
**Total no. of hospitalized adolescents**	**376 (100.0)^†^**	**204 (100.0)^†^**	**172 (100.0)^†^**
**Age, yrs, median (IQR)**	14.9 (13.4–15.9)	14.8 (13.3–15.9)	15.0 (13.5–16.0)
**Sex**			
Male	156 (41.5)	97 (47.5)	59 (34.3)
Female	220 (58.5)	107 (52.5)	113 (65.7)
**Race/Ethnicity^§^**			
Hispanic	115 (30.6)	64 (31.4)	51 (29.7)
Black, non-Hispanic	117 (31.1)	73 (35.8)	44 (25.6)
White, non-Hispanic	114 (30.3)	52 (25.5)	62 (36.0)
Asian/Pacific Islander, non-Hispanic	14 (3.7)	6 (2.9)	8 (4.7)
Persons of all other races^¶^	3 (0.8)	3 (1.5)	0 (—)
Unknown race/ethnicity	13 (3.5)	6 (2.9)	7 (4.1)
**Residence type**			
Private residence	344 (94.8)	195 (95.6)	149 (93.8)
Congregate setting, other, or unknown residence type**	19 (5.2)	9 (4.4)	10 (6.3)
**Primary reason for admission**			
Likely COVID-19-related	204 (54.3)	204 (100.0)	0 (—)
Obstetrics	21 (5.6)	0 (—)	21 (12.2)
Inpatient surgery	24 (6.4)	0 (—)	24 (14.0)
Psychiatric admission requiring medical care	76 (20.2)	0 (—)	76 (44.2)
Trauma	22 (5.9)	0 (—)	22 (12.8)
Other reason	13 (3.5)	0 (—)	13 (7.6)
Unknown reason	16 (4.3)	0 (—)	16 (9.3)
**COVID-19-related symptoms at admission^††^**			
Yes, symptomatic	259 (71.9)	192 (94.1)	67 (42.9)
**Hospitalization outcomes**			
Length of hospital stay, days, median (IQR)	2.7 (1.2–6.1)	2.4 (1.1–5.7)	3.2 (1.4–6.7)
ICU admission	93 (25.6)	64 (31.4)	29 (18.2)^§§^
Invasive mechanical ventilation	21 (5.8)	10 (4.9)	11 (6.9)^§§^
In-hospital death	0 (—)	0 (—)	0 (—)
**Underlying medical condition**			
≥1 underlying medical condition^¶¶^	207 (55.1)	144 (70.6)	63 (36.6)
Obesity***	101 (27.9)	73 (35.8)	28 (17.7)
Chronic lung disease, including asthma	87 (24.0)	63 (30.9)	24 (15.2)
Neurologic disorders	43 (11.9)	29 (14.2)	14 (8.9)
Chronic metabolic disease, including diabetes	32 (8.8)	24 (11.8)	8 (5.1)
Immunocompromised condition	20 (5.5)	14 (6.9)	6 (3.8)
Blood disorder, including sickle cell anemia	21 (5.8)	19 (9.4)	2 (1.3)
Cardiovascular disease	15 (4.1)	9 (4.4)	6 (3.8)

**Abbreviations:** COVID-NET = Coronavirus Disease 2019–Associated Hospitalization Surveillance Network; ICU = intensive care unit; IQR = interquartile range.

* Includes persons admitted to a hospital with an admission date between January 1, 2021 and March 31, 2021. Counties included in COVID-NET surveillance: California (Alameda, Contra Costa, and San Francisco counties); Colorado (Adams, Arapahoe, Denver, Douglas, and Jefferson counties); Connecticut (Middlesex and New Haven counties); Georgia (Clayton, Cobb, DeKalb, Douglas, Fulton, Gwinnett, Newton, and Rockdale counties); Iowa (one county represented); Maryland (Allegany, Anne Arundel, Baltimore, Baltimore City, Calvert, Caroline, Carroll, Cecil, Charles, Dorchester, Frederick, Garrett, Harford, Howard, Kent, Montgomery, Prince George’s, Queen Anne’s, St. Mary’s, Somerset, Talbot, Washington, Wicomico, and Worcester counties); Michigan (Clinton, Eaton, Genesee, Ingham, and Washtenaw counties); Minnesota (Anoka, Carver, Dakota, Hennepin, Ramsey, Scott, and Washington counties); New Mexico (Bernalillo, Chaves, Doña Ana, Grant, Luna, San Juan, and Santa Fe counties); New York (Albany, Columbia, Genesee, Greene, Livingston, Monroe, Montgomery, Ontario, Orleans, Rensselaer, Saratoga, Schenectady, Schoharie, Wayne, and Yates counties); Ohio (Delaware, Fairfield, Franklin, Hocking, Licking, Madison, Morrow, Perry, Pickaway and Union counties); Oregon (Clackamas, Multnomah, and Washington counties); Tennessee (Cheatham, Davidson, Dickson, Robertson, Rutherford, Sumner, Williamson, and Wilson counties); and Utah (Salt Lake County).

^†^ Data are missing for <5 percent of observations for all variables.

^§^ If ethnicity was unknown, non-Hispanic ethnicity was assumed.

^¶^ Includes non-Hispanic persons reported as other or multiple races.

** Unknown residence types are those which have not yet been ascertained from the medical chart but have not been determined to be missing.

^††^ COVID-19-related symptoms included respiratory symptoms (congested/runny nose, cough, hemoptysis/bloody sputum, shortness of breath/respiratory distress, sore throat, upper respiratory infection, influenza-like illness, wheezing) and non-respiratory symptoms (abdominal pain, altered mental status/confusion, anosmia/decreased smell, chest pain, conjunctivitis, diarrhea, dysgeusia/decreased taste, fatigue, fever/chills, headache, muscle aches/myalgias, nausea/vomiting, rash, seizures). Symptoms are abstracted from the medical chart and might not be complete.

^§§^ ICU admission and invasive mechanical ventilation are not mutually exclusive categories, and patients could have received both. Of the 31 patients whose primary reason for admission was not COVID-19-related but who were admitted to the ICU or received invasive mechanical intervention, nine (29.0%) were admitted because of trauma and 14 (45.2%) were admitted for a psychiatric admission requiring medical care, including suicide attempts or overdoses.

^¶¶^ Defined as ≥1 of the following: chronic lung disease, chronic metabolic disease, blood disorder/hemoglobinopathy, cardiovascular disease, neurologic disorder, immunocompromised condition, renal disease, gastrointestinal/liver disease, rheumatologic/autoimmune/inflammatory condition, obesity, feeding tube dependency, wheelchair dependency, or pregnancy.

*** Obesity is defined as body mass index (kg/m^2^) ≥95th percentile for age and sex based on CDC growth charts among children, a discharge diagnosis of obesity indicated by *International Classification of Diseases, Tenth Edition, Clinical Modification* (ICD-10-CM) codes, or obesity is indicated as an underlying condition on the COVID-NET case report form for adolescents who were not pregnant at the time of admission.

During March 1, 2020–April 24, 2021, the cumulative COVID-19–associated adolescent hospitalization rate (49.9) was 12.5 times lower than that in adults aged ≥18 years (675.6). Weekly COVID-19–associated adolescent hospitalization rates (3-week moving average) were comparable to rates among those aged 0–4 years, but higher than rates among children aged 5–11 years (Figure 1). Weekly adolescent hospitalization rates peaked at 2.1 per 100,000 during the week ending January 9, 2021, declined to 0.6 during the week ending March 13, 2021, then increased to 1.3 and 1.2 for the weeks ending April 17 and 24, 2021, respectively. Rates among adolescents in two of 14 sites (Maryland and Michigan) were highest during April 2021 compared with all other weeks within their respective sites since surveillance began on March 1, 2020. Cumulative COVID-19–associated hospitalization rates during October 1, 2020–April 24, 2021, were 2.5–3.0 times higher than seasonal influenza-associated hospitalization rates during three recent influenza seasons (October 1–April 30) (Figure 2).

**FIGURE 1:**
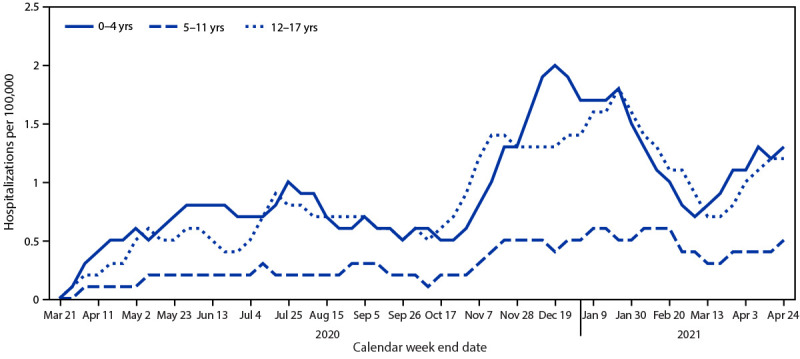
Three-week moving average COVID-19–associated hospitalization rates* among children and adolescents aged <18 years, by age group — COVID-NET, 14 states,† March 1, 2020–April 24, 2021 **Abbreviation:** COVID-NET = Coronavirus Disease 2019–Associated Hospitalization Surveillance Network. * Number of patients with laboratory-confirmed COVID-19–associated hospitalizations per 100,000 population. ^†^ COVID-NET sites are in the following 14 states: California, Colorado, Connecticut, Georgia, Iowa, Maryland, Michigan, Minnesota, New Mexico, New York, Ohio, Oregon, Tennessee, and Utah.

**FIGURE 2:**
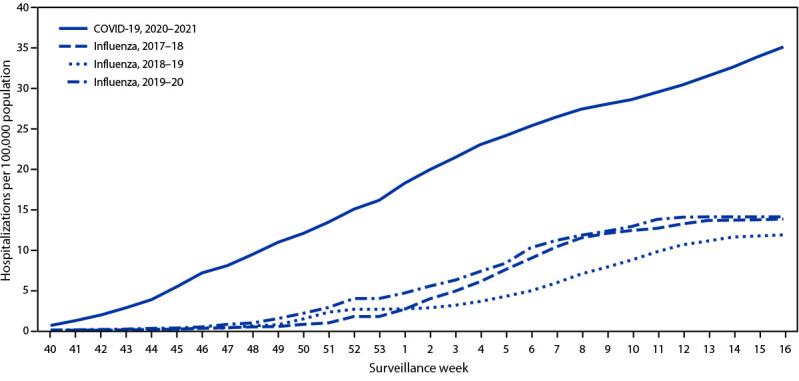
Cumulative rates for COVID-19–associated hospitalizations* compared with influenza-associated hospitalizations^†^ among adolescents aged 12–17 years, by surveillance week^§^ — COVID-NET^¶^ and FluSurv-NET,** 14 states,^††^ 2017–2021^§§^ **Abbreviations:** COVID-NET = Coronavirus Disease 2019–Associated Hospitalization Surveillance Network; FluSurv-NET = Influenza Hospitalization Surveillance Network. * Number of patients with laboratory-confirmed COVID-19-associated hospitalizations per 100,000 population. ^†^ Number of patients with laboratory-confirmed influenza-associated hospitalizations per 100,000 population. ^§^ Surveillance week is based on the epidemiologic week for disease reporting and lasts Sundays through Saturdays. MMWR week numbering is sequential beginning with 1 and incrementing with each week to a maximum of 52 or 53. The three influenza seasons had no surveillance week 53, so values from surveillance week 52 were imputed to surveillance week 53. https://wwwn.cdc.gov/nndss/document/MMWR_week_overview.pdf ^¶^ COVID-NET is a population-based surveillance system of laboratory-confirmed COVID-19–associated hospitalizations in 99 counties across 14 states. COVID-19–associated hospitalizations among residents in a predefined surveillance catchment area who received a positive test for SARS-CoV-2 (the virus that causes COVID-19) during hospitalization or ≤14 days before admission are included in surveillance. ** FluSurv-NET is a population-based surveillance system of laboratory-confirmed influenza-associated hospitalizations in 81 counties across 13 states (for the period included) and is conducted annually during October 1–April 30. Influenza-associated hospitalizations among residents in a predefined surveillance catchment area who received a positive test for influenza during hospitalization or ≤14 days before admission are included in surveillance. ^††^ COVID-NET and FluSurv-NET sites were in the following 14 states for the period shown: California, Colorado, Connecticut, Georgia, Iowa (COVID-NET only), Maryland, Michigan, Minnesota, New Mexico, New York, Ohio, Oregon, Tennessee, and Utah. ^§§^ Cumulative COVID-19–associated hospitalization rates among adolescents aged 12–17 years during October 1, 2020–April 24, 2021, were compared with influenza-associated hospitalization rates in the same age group during October 1–April 30 across three seasons (2017–18, 2018–19, and 2019–20) using data from FluSurv-NET.

## Discussion

COVID-NET data indicate that COVID-19–associated hospitalization rates were lower in adolescents aged 12–17 years compared with those in adults but exceeded those among children aged 5–11 years during March 1, 2020–April 24, 2021. Moreover, COVID-19–associated hospitalization rates among adolescents increased during March–April 2021, and nearly one third of 204 recently hospitalized adolescents required ICU admission. Rates of COVID-19–associated hospitalization among adolescents also exceeded historical rates of seasonal influenza-associated hospitalization during comparable periods. Recent increased hospitalization rates and the potential for severe disease reinforce the importance of continued COVID-19 prevention measures among adolescents, including vaccination and correct and consistent wearing of masks.

After declines in January and February 2021, weekly population-based rates of COVID-19–associated hospitalization among adolescents increased during March and April, and in two COVID-NET sites (Maryland and Michigan) the highest adolescent COVID-19–associated hospitalization rates in their respective sites since the start of the COVID-19 pandemic occurred during this period. This trend contrasts with hospitalization rates among persons aged ≥65 years, the group with the highest COVID-19 vaccination coverage, among whom hospitalization rates in COVID-NET stabilized during the same period.^¶¶^ Increased hospitalization rates among adolescents might be related, in part, to circulation of particularly transmissible SARS-CoV-2 variants,*** the larger numbers of children returning to school or other in-person indoor activities, and changes in physical distancing, wearing masks, and other COVID-19 prevention behaviors (*4*). SARS-CoV-2 transmission occurs more easily in high schools than in elementary schools (*4*), and outbreaks have been associated with high school extracurricular activities (*5*). Vaccination of adolescents is expected to reduce the risk for COVID-19 in these settings.

Population-based COVID-19–associated hospitalization rates among adolescents were lower than were those in adults, a finding consistent with studies showing that illness is generally milder in children than in adults (*6*). Nevertheless, severe disease does occur, including that requiring ICU admission and invasive mechanical ventilation. Most (70.6%) adolescents in this study whose primary reason for hospitalization was COVID-19–associated illness had at least one underlying medical condition, which is lower than the percentage of hospitalized adults with an underlying medical condition (92%) (*7*). Nearly 30% of these adolescents had no reported underlying medical condition, indicating that healthy adolescents are also at risk for severe COVID-19–associated disease. In addition, approximately two thirds of adolescents hospitalized with COVID-19 were Hispanic or non-Hispanic Black persons, consistent with studies showing an increased incidence of COVID-19 among racial and ethnic minority populations and signifying an urgent need to ensure equitable access to vaccines for these groups (*8*). Vaccination is effective in preventing hospitalization among adults (*9*); similarly, widespread vaccination of adolescents will likely reduce COVID-19–associated hospitalizations, and potential sequelae from COVID-19 in adolescents, including multisystem inflammatory syndrome in children (MIS-C), a rare but serious complication of COVID-19 (*10*).

During a comparable period, adolescent hospitalization rates associated with COVID-19 exceeded those for seasonal influenza, another respiratory virus that can cause hospitalization and death in adolescents and for which a vaccine is recommended in this age group.^†††^ This widespread circulation of SARS-CoV-2 occurred despite containment measures such as school closures, wearing masks, and physical distancing, none of which had been enacted during the historical influenza seasons. Without these containment measures, the rates of COVID-19–associated hospitalization might have been substantially higher.

The findings in this report are subject to at least five limitations. First, the primary reason for hospital admission was not always clear, and some (45.7%) adolescents who met the COVID-NET case definition were hospitalized for reasons that might not have been primarily related to COVID-19, despite a positive SARS-CoV-2 laboratory test result; these hospitalizations were included in rate calculations. Thus, rates of hospitalizations for COVID-19 might be overestimated. Second, laboratory confirmation depends on clinician-ordered testing and hospital testing policies for SARS-CoV-2 (COVID-NET) and influenza (FluSurv-NET); consequently, hospitalization rates might also be underestimated. Given more widespread testing for SARS-CoV-2 compared with influenza, the lack of adjustment for testing practices likely disproportionately affects influenza rates compared with COVID-19 rates. Third, adolescents hospitalized with MIS-C might not be identified if testing occurred >14 days before admission, potentially leading to an underestimate of severe COVID-19–associated disease. Fourth, the Pfizer-BioNTech COVID-19 vaccine had been approved for and administered to adolescents aged 16–17 years during this study period; therefore, rates of COVID-19–associated hospitalization in adolescents aged 16–17 years might differ from those in adolescents aged 12–15 years who were not previously eligible for vaccination, and could affect the overall hospitalization rate for all adolescents. Finally, hospitalization rates are preliminary and might change as additional data are reported.

Recent increases in COVID-19–associated hospitalization rates and the potential for severe disease requiring ICU admission, including invasive mechanical ventilation, among adolescents indicate an urgent need for vaccination in combination with correct and consistent mask wearing by persons not yet fully vaccinated or when required by laws, rules, or regulations. Highly effective COVID-19 vaccines are now available to adolescents as an additional evidence-based prevention measure (*9*); expansion of COVID-19 vaccination of adolescents, with particular attention to racial and ethnic minority groups disproportionately affected by severe COVID-19, is expected to reduce COVID-19–associated morbidity within this age group.

**Summary**

**What is already known about this topic?**

Most COVID-19–associated hospitalizations occur in adults, but severe disease occurs in all age groups, including adolescents aged 12–17 years.

**What is added by this report?**

COVID-19 adolescent hospitalization rates from COVID-NET peaked at 2.1 per 100,000 in early January 2021, declined to 0.6 in mid-March, and rose to 1.3 in April. Among hospitalized adolescents, nearly one third required intensive care unit admission, and 5% required invasive mechanical ventilation; no associated deaths occurred.

**What are the implications for public health practice?**

Recent increased hospitalization rates in spring 2021 and potential for severe disease reinforce the importance of continued COVID-19 prevention measures, including vaccination and correct and consistent mask wearing among persons not fully vaccinated or when required.

## Footnotes

* https://www.fda.gov/news-events/press-announcements/coronavirus-covid-19-update-fda-authorizes-pfizer-biontech-covid-19-vaccine-emergency-use; https://www.cdc.gov/media/releases/2021/s0512-advisory-committee-signing.html

†
https://www.cdc.gov/coronavirus/2019-ncov/vaccines/fully-vaccinated.html

§ California, Colorado, Connecticut, Georgia, Iowa, Maryland, Michigan, Minnesota, New Mexico, New York, Ohio, Oregon, Tennessee, and Utah.

¶
https://www.cdc.gov/coronavirus/2019-ncov/covid-data/covid-net/purpose-methods.html

** Those excluded were patients admitted for 1) labor and delivery (pregnant) (5.6%), 2) inpatient procedures/surgery (6.4%), 3) psychiatric reasons but requiring medical care (20.2%), 4) trauma (5.9%), and 5) other or unknown reasons (7.8%) with no recorded COVID-19–associated symptoms upon admission and who might have been identified through routine testing upon admission.

†† FluSurv-NET, which has similar methods for case ascertainment and catchment areas as COVID-NET, conducts seasonal laboratory-confirmed influenza-associated hospitalization surveillance during October 1–April 30 annually.

§§ 45 C.F.R. part 46, 21 C.F.R. part 56; 42 U.S.C. Sect. 241(d); 5 U.S.C. Sect. 552a; 44 U.S.C. Sect. 3501 et seq.

¶¶
https://gis.cdc.gov/grasp/COVIDNet/COVID19_3.html

***
https://covid.cdc.gov/covid-data-tracker/#variant-proportions

†††
https://www.cdc.gov/mmwr/volumes/69/rr/rr6908a1.htm
